# Case-mix fails to explain variation in mastectomy rates: management of screen-detected breast cancer in a UK region 1997–2003

**DOI:** 10.1038/sj.bjc.6602264

**Published:** 2004-12-21

**Authors:** L J M Caldon, S J Walters, J A Reed, A Murphy, A Worley, M W R Reed

**Affiliations:** 1 Academic Surgical Oncology Unit, Division of Surgical Sciences (South), Section of Surgical & Anaesthetic Sciences, The University of Sheffield, Floor K, Royal Hallamshire Hospital, Sheffield S10 2JF, UK; 2 School of Health and Related Research, Sheffield Health Economics Group, SHARR, University of Sheffield, Regent Court, 30 Regent St, Sheffield S1 4DA, UK; 3 East Midlands Breast Screening Quality Assurance Reference Centre, Rufford Ward, Nottingham City Hospital NHS Trust, Hucknall Road, Nottingham NG5 1PB, UK

**Keywords:** breast cancer, mastectomy, screening, audit, variation, case-mix adjustment

## Abstract

Wide variation in the surgical management of breast cancer exists at hospital, regional, national and international level. To demonstrate whether variation in surgical practice observed at aggregate level between breast units persists following adjustment for case-mix, individual patient-level data from the Trent Breast Screening Programme Quality Assurance database (1997–2003) was analysed. Expected case-mix adjusted mastectomy rates were derived by logistic regression using the variables tumour size, site and grade, patient age and year of presentation, employing the region's overall case-mix adjusted practice as the reference population. The region's 11 breast screening units detected 5109 (3989 invasive) surgically managed primary breast cancers over the 6-year period. A total of 1828 mastectomies (Mx) were performed (Mx rate 35.8%, 95% confidence interval: 34.5–37.1%). Significant variation in mastectomy rates were observed between units (range 25–45%, *P*<0.0001), and persists following case-mix adjustment (*P*<0.0001). Two-fold variation in observed to expected unit mastectomy rate coefficient is demonstrated overall (range 0.66–1.36), increasing to almost four-fold variation in cancers less than 15 mm diameter (range 0.55–1.95). Significant variation in surgery for screen-detected primary breast cancer is not explained by case-mix. Further research is required to investigate potential patient and professional causative factors.

Over the last 10 years, evidence of long-term randomised trials of the surgical management of early (stage 1 and 2) breast cancer have conclusively demonstrated equivalent survival rates in women with cancers up to 40 and 50 mm diameter, when treated with either mastectomy or breast-conservation surgery with ipsilateral radiotherapy (van Dongen *et al*, 2000; Fisher *et al*, 2002a, 2002b). Over the same period, evidence has emerged that inclusion of patients in decisions about their health is associated with improved physical and psychological outcomes (Wolberg, 1990; Fallowfield *et al*, 1994; Stewart, 1995). Such knowledge led to a paradigm shift from the view of the patient as ‘a passive recipient of medical intervention‘, to a client or partner (Rawling, 1992), and the empowerment of patients in the decision-making process in health care (Wolberg, 1990; Richards *et al*, 1995; Stewart, 1995; Charles *et al*, 1999; Royal College of Surgeons of England, 2002). Consequently, it was widely assumed that when offered a choice of surgery, the majority of women would elect to undergo breast-conservation surgery. Evidence suggests this has not been the case.

Variability in the surgical management of early breast cancer has long been observed and reported at breast unit/hospital level, and regional/state level, both within the UK and internationally (Iscoe *et al*, 1994; Goel *et al*, 1997; Morrow *et al*, 2001; Sauven *et al*, 2003). The issue of geographic variation in mastectomy rates has been highlighted in recent UK Department of Health documents, where practice variation is referred to as a ‘post code lottery’ of cancer management, and the concept of using the mastectomy to lumpectomy ratio has been introduced as a performance indicator of breast unit practice (Department of Health, 2000, 2001). The use of raw treatment rates, unadjusted for the case-mix, has the potential for providing a misleading impression of practice, especially if based on small numbers of treated individuals.

The majority of published studies on the issue conclude that significant variation in treatment exists, which cannot be explained by case-mix alone (Nattinger and Goodwin, 1994; Iscoe *et al*, 1994; Goel *et al*, 1997). However, these conclusions are based on aggregated data analysis, which fail to take account of case-mix.

Trent was until recently one of the eight National Health Service regions of England and Wales. Situated geographically in the centre and east of England, it has a population of around 5 million, approximately 2 500 000 females (2001 Census, 2004). The UK National Health Service Breast Screening Programme (NHSBSP) set up in 1988 invites all women aged 50–64 years to attend routine mammography. The population eligible for screening by the Trent Breast Screening Programme was 441 000.

Breast unit and regional guidelines exist for the management of breast cancer in the UK, but vary as there are no specific national guidelines on the surgical management of the disease. Each unit in Trent has a set of treatment guidelines, which include indications and relative indications for the different surgical options; for example, indications for mastectomy are patient choice, tumour size (typically >40 mm diameter clinical size), multifocal disease, and contraindication to radiotherapy. Relative indications for mastectomy are lobular carcinoma, multicentric carcinoma within a single breast quadrant, central tumour; breast conservation likely to result in an unacceptable aesthetic outcome, and breast-conservation surgery where radiotherapy is likely to be associated with high risk of complications. Guidelines often contain the postscript that larger tumours may be managed by breast conservation under certain circumstances; for example, where tumour to breast size ratio permits an acceptable outcome in terms of aesthetic result, patient survival and local recurrence risk. It should be emphasised that such guidelines are designed to be permissive; their content intended to promote sufficient flexibility within the confines of known evidence, to facilitate optimal collaboration in the decision-making process between patients and their treating professionals.

The aim of this study was to demonstrate whether variation in surgical practice observed at aggregate level between units over the Trent region, reported in Trent Breast Screening Programme annual reports (Reed, 2004), persisted following adjustment for the characteristics of cases managed by the individual units.

## METHODS

This observational study analyses quality assurance data collected by the Trent Breast Screening Programme on women diagnosed with breast cancer as part of the UK NHSBSP between April 1997 and April 2003. Individual patient-level data from the database was anonymised prior to conversion into an SPSS data file. The password-protected database and outputs were stored securely.

Since the main outcome of treatment data was binary, that is, whether or not the woman had a mastectomy, multiple logistic regression with SPSS for windows version 12 was employed to analyse the dataset at individual patient level to confirm whether observed unit level variations persist following adjustment for case-mix (tumour size, site, patient age) and year of treatment. For the purposes of this study, maximum tumour size was defined as the greatest recorded diameter (invasive or noninvasive) where tumours comprised both invasive and noninvasive components. Year of treatment was included as a variable to reflect changes in evidence-based practice over time. Age, maximum tumour size and year of diagnosis were treated as continuous covariates. Tumour grade was categorised into invasive grade 1, 2 and 3, and noninvasive, and tumour site grouped into central and noncentral.

Applying the logistic model at an individual patient level, the individuals’ probability of undergoing a mastectomy was calculated given their clinical covariates. Expected individual screening unit mastectomy rates were calculated by the summation of individual patient level probabilities of undergoing a mastectomy across that particular unit. The ratio of observed to expected mastectomies (Mx) for each breast-screening unit were then calculated. Observed to expected ratios above 1.0 suggest that after adjustment for case-mix, the unit has a higher than expected Mx rate compared to the average (using overall case-mix adjusted practice in Trent as the reference population). Conversely, observed to expected ratios below 1.0 suggest a lower than expected Mx rate compared to the average.

Case-mix adjustment was performed twice. The first analysis incorporated the variables tumour size, tumour site, patient age and year of screening. The second included tumour grade in addition. The first analysis reflects information uniformly available prior to surgical decision-making in all the region's units, and thus the variables upon which operative advice and decision-making are based. Tumour grade is documented preoperatively in a proportion of cases, and may influence guideline-based treatment decision-making in certain units.

## RESULTS

During the period April 1997 to April 2003, 792 570 women were screened by Trent's 11 Breast Screening Units, incorporating 13 static sites and nine mobile diagnostic units. Over the 6-year period, 5179 primary breast cancers were diagnosed through the programme, 5109 (3989 invasive) were managed surgically, 70 did not undergo surgery. Advanced disease or ‘other clinical factors’ were stated as the reason for not undergoing surgery in the majority of cases.

Breast units' performance over the 6-year period was aggregated. Annual fluctuations were inherent within most services.

Table 1 describes the characteristics of the tumours detected by the Trent Breast Screening Program over the 6-year period. Between 1997 and 2003, 1828 Mx were performed, giving an overall Trent region mastectomy rate of 35.8% (95% confidence interval (CI): 34.5–37.1%).

Table 2 illustrates observed unit mastectomy rates and those expected following case-mix adjustments for all cancers (*n*=5060). Significant variation in mastectomy rates is illustrated across the 11 units, with individual unit Mx rates ranging from 25 to 45% (*P*<0.0001). Expected rates derived by logistic regression, using the region's overall case-mix adjusted practice as the reference population, demonstrate a two-fold variation in ratio of observed to expected unit mastectomy rate (range 0.66–1.36). Units 6 and 10 demonstrate statistically significant lower (34%) and higher (36%) than expected rates, respectively. In addition, Unit 4 exhibits a 13% higher observed to expected mastectomy rate ratio, although this just fails to reach statistical significance.

When case-mix data analysis was repeated including the tumour grade variable, 105 out of 5109 (2%) cases were excluded due to missing or incomplete data. Comparison of the results of the two forms of data analysis revealed no difference in the ratio of observed to expected unit mastectomy rates when case-mix adjustment included or excluded tumour grade as a variable. Thus, the observed variation in mastectomy rates across the 11 screening units demonstrated in Trent cannot be accounted for by tumour size, site or grade, patient age or year of screening.

**Figure 1:**
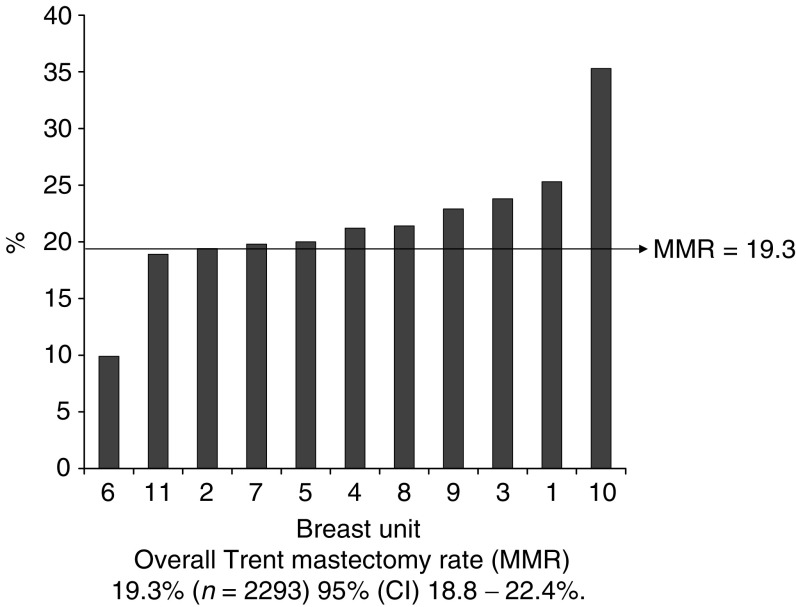
Unit mastectomy rates for small (total tumour size less than 15 mm diameter), invasive and noninvasive screen-detected cancers in the Trent region, 1997–2003. Overall Trent mastectomy (MMR) 19.3% (*n*=2293), 95% (CI) 18.8–22.4.

In total, 46% (2329 out of 5062) of the women included in the study had small tumours (less than 15 mm diameter). The overall Mx rate in this subgroup was 19.3% (442 out of 2293); 95% CI: 17.9–21.1%, and again varied significantly (*P*<0.0001) across the 11 units from 10 to 35%. Within the subgroup, an almost four-fold variation in observed to expected mastectomy rate ratio between breast units is demonstrated (range 0.55–1.95). Table 3 and Figure 1 demonstrate variation in mastectomy rates for small (less than 15 mm diameter) tumours.

## DISCUSSION

This observational study of a single UK region's breast-screening practice demonstrates statistically significant treatment variation in the surgical management of early stage breast cancer. The study's strength lies in the analysis of patient data at an individual level. By correcting for case-mix and comparing observed and case-mix adjusted Mx rates, a unit's mastectomy rate is effectively adjusted for any variation in the type of cases presenting to them. To the authors knowledge this is the first study of this type demonstrating persistent breast cancer surgical treatment variation. The study demonstrates that variables other than those included in the case-mix adjustment (tumour size, tumour site, tumour grade, patient age and year of presentation) are responsible for the variation observed.

The conclusions drawn from this study are based purely on breast cancers detected by the breast-screening programme and do not include those diagnosed through the symptomatic breast service. Thus, the data analysis only accurately reflects unit practice in the screen-detected subgroup, where the majority of women (82%) are aged 50–64 years and have relatively small tumours. The interunit variation in Mx rate demonstrated by this study may be atypical of women with breast cancer, reflecting variability only within the screened subgroup. Further research is needed to determine whether or not unit variation exists in the non-screen-detected population.

There are several potential limitations to this type of study and analysis. One is the use of an inappropriate data set with large amounts of missing data. We believe the study described uses robust data; the data has been rigorously audited by the quality assurance service and validated both externally and by the surgeons of the originating breast units. In areas audited by the study, the data was 98% complete; 109 cases (2%) excluded from analysis due to missing or incomplete data.

Overall tumour size as determined histologically was used in the analysis, rather than radiological tumour size. Evidence suggests histological size and radiological tumour size correlate well, with good reliability of radiological assessment of tumour size based on ultrasound and mammography (Pain *et al*, 1992).

The present study employed overall Trent screen-detected breast cancer population (1997–2003) as the reference population. An argument could be made that the reference population should be the overall treated UK NHSBSP population. This would require access to individual level data of the entire treated UK screen-detected population, and though the absolute level of observed to expected coefficients may have altered, the degree of variation between units' coefficients would not.

Another potential limitation of the study is that observations are based on the data of a single UK region. Trent as a region and its units may be atypical of other UK regions and their units. There is, however, evidence that such variation in Mx rates occurs throughout the UK at regional and unit level, in symptomatic and screen-detected breast cancer practice, to a similar degree as that identified in Trent (Sauven *et al*, 2003; Moneypenny, 2004a; NHS Cancer Screening Programmes and Association of Surgeons at BASO, 4 A.D., 2004).

The study concentrated on a small number of tumour characteristics and patient's age. Screening year was included as a proxy for time changes in evidence-based practice. It is recognised that other clinical factors, not included in the study's case-mix adjustment analysis, could fully or partially explain the pattern of treatment variation observed. Variables included in the analysis were chosen to reflect information routinely available at the time of decision-making.

The analysis performed is based purely on the information contained within an existing database; patients were not contacted for the purposes of this study. Therefore, certain other variables of interest, such as such as tumour to breast size ratio, radiological tumour size and information on educational level, family income and decision-making style, not recorded on the database, are unavailable. Data such as postcode, which could have provided a surrogate measure of socioeconomic profile and permit assessment of whether distance of patient's home to radiotherapy treatment centre influences treatment rates, were not available because data were anonymised at source.

The study was conducted on a screen-detected population where approximately 85% of women diagnosed with primary breast cancer had tumours less than 30 mm diameter. On the basis of tumour size alone, the majority of women in this group were eligible for a choice of treatment. Within the subgroup with small tumours (total tumour size less than 15 mm diameter), there is an almost four-fold variation in observed to expected coefficients following case-mix adjustment. This finding concords with data from another UK study by Sauven *et al* (2003) illustrating a similar degree of variation in treatment at regional level in the UK in the screen-detected subgroup with small tumours. The study by Sauven, however, failed to adjust for case-mix.

Although the study does not include information on the non-screen-detected breast cancer population, it is probable that symptomatic and screening practice is similar as patients are treated by the same team of health care professionals. The results of a recent audit, presented at the annual meeting of the Association of Breast Surgery at the British Association of Surgical Oncology on 26th May 2004, demonstrate a similar pattern of treatment variation at breast unit level for cancers presenting symptomatically to 50 breast units in the UK over a 6–12-month period from April 2002 to 2003 (Moneypenny, 2004b).

The explanation of wide variation in mastectomy rates remains unclear from both our study and available literature. Research is needed to investigate the potential factors influencing choice of surgery in breast cancer. The process involved in decision-making with respect to surgery in this disease is complex; the final decision being the result of primary tumour characteristics and communication between, and interaction of, multiple individuals each with their own pre-existing characteristics and influences interacting with each other over a series of encounters. Future studies to elucidate surgical treatment variation in breast cancer need to focus on the influence of both potential patient and professional (specialist surgeon and nurse) factors on decision-making.

## Figures and Tables

**Table 1:** Clinical characteristics of Trent breast-screening programme quality assurance database patients, 1997–2003 (*n*=5109)

	**Cases (*n*)**	**Mean**	**Median**	**Std deviation**	**Minimum**	**Maximum**
Age at diagnosis (years)	5109	59.1	58.8	6.2	45.3	95.0
Maximum tumour size (mm)	5062	18.5	15.0	14.3	0.5	220.0
					***n***	**%**
Year (financial)	1997/1998				652	12.8
	1998/1999				802	15.7
	1999/2000				799	15.6
	2000/2001				917	17.9
	2001/2002				866	17.0
	2002/3				1073	21.0
Total (*n*)					5109	100
					
Overall type of surgery	Conservation				3281	64.2
	Mastectomy				1828	35.8
Total (*n*)					5109	100
					
Invasive status	Invasive and mixed				3989	78.1
	Noninvasive only				1120	21.9
Total (*n*)					5109	100
					
Tumour size (mm)	<15				2329	46.0
	⩾15–<20				995	19.7
	⩾20–<30				1060	20.9
	⩾30–<50				480	9.5
	⩾50				198	3.9
Total (*n*)					5062	100
					
Tumour site	Central/nipple region				288	5.6
	Noncentral				4818	94.5
Total (*n*)					5106	100
					
Tumour grade	Noninvasive				1120	22.2
	Invasive grade 1				1209	23.9
	Invasive grade 2				1870	37.0
	Invasive grade 3				854	16.9
Total (*n*)					5053	100

**Table 2:** Observed *vs* expected mastectomies (Mx) by screening unit 1997–2003 (all cancers)

**Unit**	**Total cancers (*n*)**	**Observed (O) Mx (*n*)**	**Observed (O) Mx (%) Rate (%)**	**Expected (E) Mx (*n*)**	**Ratio O/E**	**(95% CI)**
1	209	89	42.6	77	1.15	(0.93–1.42)
2	310	106	34.2	114	0.93	(0.76–1.12)
3	415	159	38.3	138	1.15	(0.98–1.35)
4	723	250	34.6	221	1.13	(1.00–1.28)
5	367	148	40.3	139	1.06	(0.90–1.25)
6	840	213	25.4	321	0.66	(0.58–0.76)
7	345	118	34.2	124	0.95	(0.79–1.14)
8	253	94	37.2	79	1.19	(0.96–1.46)
9	916	390	42.6	367	1.06	(0.96–1.17)
10	235	106	45.1	78	1.36	(1.11–1.64)
11	447	140	31.3	155	0.90	(0.76–1.06)
						
Trent	5060	1813	35.8	1813	1.00	

The expected numbers at each screening unit are based on adjusting each unit's case-mix for age, tumour site, tumour size, year of screening. A total of 49 patients have been excluded due to missing data.

**Table 3:** Observed *vs* expected mastectomies (Mx) by screening unit, 1997–2003

**Unit**	**Total cancers (*n*)**	**Observed (O) Mx (*n*)**	**Observed (O) Mx (%) Rate (%)**	**Expected (E) Mx (*n*)**	**Ratio O/E**	**(95% CI)**
1	96	24	25.0	20	1.22	(0.78–1.82)
2	148	26	17.6	30	0.86	(0.56–1.26)
3	192	40	20.8	35	1.14	(0.82–1.56)
4	417	82	19.7	79	1.03	(0.82–1.28)
5	136	28	20.6	25	1.13	(0.75–1.64)
6	323	33	10.2	60	0.55	(0.38–0.77)
7	142	27	19.0	27	0.99	(0.65–1.14)
8	131	27	20.6	26	1.06	(0.70–1.54)
9	405	87	21.5	83	1.05	(0.84–1.29)
10	111	38	34.2	20	1.95	(1.38–2.67)
11	192	30	15.6	37	0.80	(0.54–1.14)
Trent	2293	442	19.3	442	1.00	

Tumours less than 15 mm in diameter.

The expected numbers at each screening unit are based on adjusting each unit's case-mix for age, tumour site, tumour grade and year of screening. A total of 36 patients have been excluded due to missing data.
